# The value of systemic inflammatory markers in identifying malignancy in mucinous pancreatic cystic neoplasms

**DOI:** 10.18632/oncotarget.23310

**Published:** 2017-12-14

**Authors:** Wentao Zhou, Yefei Rong, Tiantao Kuang, Yadong Xu, Xiaojing Shen, Yuan Ji, Wenhui Lou, Dansong Wang

**Affiliations:** ^1^ Department of General Surgery, Zhong Shan Hospital, Fudan University, Shanghai, China; ^2^ Department of Pancreatic Surgery, Zhong Shan Hospital, Fudan University, Shanghai, China; ^3^ Department of Pathology, Zhong Shan Hospital, Fudan University, Shanghai, China

**Keywords:** systemic inflammatory markers, pancreatic cystic neoplasm, invasive carcinoma, platelet-to-lymphocyte ratio, lymphocyte-to-monocyte ratio

## Abstract

The treatment decision-making of mucinous pancreatic cystic neoplasm (PCN) has become a common clinical problem since the diagnostic accuracy of current tests in identifying malignancies in pancreatic cysts is limited. In this study, we aimed to validate the predictive value of systemic inflammatory factors in detecting malignant PCNs. Two hundred and forty-five patients with pathologically confirmed mucinous PCNs in a single Chinese institution were retrospectively analyzed. Receiver operating characteristic (ROC) curves were calculated to determine the optimal cut-off values and measure the diagnostic value. The results showed that neutrophil count (*P* = 0.009), lymphocyte count (*P* = 0.002), neutrophil-to-lymphocyte ratio (NLR, *P* < 0.001), platelet-to-lymphocyte ratio (PLR, *P* < 0.001) and lymphocyte-to-monocyte ratio (LMR, *P* < 0.001) were distributed differently among the various differentiation groups of PCN. The univariate analyses indicated that a neutrophil count ≥ 2.8 × 10^9^/L (*P* = 0.024), lymphocyte count ≤ 1.9 × 10^9^/L (*P* < 0.001), PLR ≥ 125 (*P* < 0.001), NLR ≥ 1.96 (*P* < 0.001), and LMR ≤ 4.29 (*P* < 0.001) were significantly associated with invasive carcinomas in PCN patients. In addition, the multivariate analyses demonstrated that PLR ≥ 125 and LMR ≤ 4.29 were independent predictors of invasive malignancies. The ROC curves exhibited the malignant detection utility of the independent factor-based predictive model with an area under the curve (AUC) of 0.858 (*P* < 0.001). In conclusion, systemic inflammatory markers provide a supportive and easily accessible tool for the preoperative diagnoses of malignant PCNs.

## INTRODUCTION

Mucin-producing pancreatic cystic neoplasms (PCNs) are precancerous lesions and comprise intraductal papillary mucinous neoplasm (IPMN) and mucinous cystic neoplasm (MCN). With an increasingly incidental detection using advanced cross-sectional imaging, the management of mucinous PCNs has gradually become a common clinical conundrum of balancing the risk of malignant transformation with the morbidity and mortality of surgical resection [1]. The Sendai consensus guidelines and subsequent updated Fukuoka consensus guidelines have been widely adopted as an algorithm to direct the treatment strategies for mucinous PCN patients [2, 3]. The high-risk parameters for malignancy in the guidelines were verified by several retrospective studies, but the outcomes have been controversial [4, 5]. Some articles reported that many mucinous PCNs did not meet these criteria and were still proven to harbor high-grade dysplasia or even invasive cancers, and the validity of the guidelines were doubtful [6, 7]. Thus, novel and accurate diagnostic indexes are required to discriminate benign lesions from invasive malignancies.

The relationship between inflammation and a tumor has been researched for more than 100 years, when it was first proposed by Virchow [8]. Accumulating evidence indicates that systemic inflammation plays a pivotal role in the initiation and progression of malignant tumors via the release of cytokines and other mediators [9–12]. Several circulating blood-cell-based markers, such as the neutrophil-to-lymphocyte ratio (NLR), platelet-to-lymphocyte ratio (PLR), and lymphocyte-to-monocyte ratio (LMR), are reflections of host systemic responses to malignancies and have been validated as independent prognostic factors in various solid tumors [13–16]. Recently, the malignancy-predicting role of preoperative NLR and PLR in PCN patients has gained much attention [1, 17].

This study was designed to assess the utility of inflammatory markers in identifying invasive carcinomas in a cohort of all mucinous PCN patients, which is difficult to distinguish using radiological images due to the overlapping morphological features between IPMN and MCN.

## RESULTS

A total of 245 patients with mucinous pancreatic cystic neoplasms were enrolled into this retrospective study. The present cohort was comprised of 149 females and 96 males with a median age of 58 years (IQR 48-65 years). Nearly half (n = 116, 47.3%) of the patients presented with symptoms, and most of these were epigastric discomfort. The majority (n = 120, 49.0%) of the patients underwent distal pancreatectomy, and 14 of them had the spleen preserved. Other surgical procedures included pancreatoduodenectomy (n = 89, 36.3%), middle segmentectomy (n = 12, 4.9%), enucleation (n = 16, 6.5%) and total pancreatectomy (n = 8, 3.3%). There were 162 (66.1%) IPMN cases and 83 (33.9%) MCN cases confirmed by pathology. The IPMNs were further classified as subtypes of the main duct (n = 55, 34.0%), branch duct (n = 58, 35.8%) and mixed type (n = 35, 21.6%). Additional clinicopathological characteristics are detailed in Table 1.

**Table 1:** Clinicopathological characteristics of resected patients with mucinous pancreatic cystic neoplasms

Characteristics	n = 245
Age, years (median, IQR)	58 (48-65)
Sex (n, %)	
Female	149 (60.8%)
Male	96 (39.2%)
Symptom, present (n, %)	116 (47.3%)
Tumor size, mm (median, IQR)	35 (20-54)
Main pancreatic duct diameter ≥ 10 mm (n, %)	28 (11.4%)
Surgical style (n, %)	
Pancreatoduodenectomy	89 (36.3%)
Distal pancreatectomy/spleen preserved	120 (49.0%)/14 (11.7%)
Total pancreatectomy	8 (3.3%)
Middle segmentectomy	12 (4.9%)
Enucleation	16 (6.5%)
Harvested lymph nodes, n (median, IQR)	4 (0-8)
Pathology (n, %)	
Intraductal papillary mucinous neoplasm	162 (66.1%)
Main duct	55 (34.0%)
Mixed type	35 (21.6%)
Branch duct	58 (35.8%)
Unknown	14 (8.6%)
Mucinous cystic neoplasm	83 (33.9%)
Albumin, g/L (median, IQR)	40 (38-42)
CA19-9, U/mL (median, IQR)	12.6 (7.0-25.1)
Unknown (n, %)	10 (4.1%)
CEA, ng/mL (median, IQR)	2.0 (1.3-3.2)
Unknown (n, %)	11 (4.5%)
Platelet count, × 10^9^/L (median, IQR)	199 (164-236)
White blood cell count, × 10^9^/L (median, IQR)	5.3 (4.5-6.1)
Neutrophil count, × 10^9^/L (median, IQR)	2.9 (2.2-3.6)
Lymphocyte count, × 10^9^/L (median, IQR)	1.8 (1.5-2.1)
Monocyte count, × 10^9^/L (median, IQR)	0.36 (0.29-0.47)
Platelet-to-lymphocyte ratio (median, IQR)	110.0 (92.6-141.7)
Neutrophil-to-lymphocyte ratio (median, IQR)	1.61 (1.21-2.13)
Lymphocyte-to-monocyte ratio (median, IQR)	4.88 (3.70-6.35)

CA19-9, carbohydrate antigen 19-9; CEA, carcinoembryonic antigen; IQR, interquartile range.

### The distribution of inflammatory markers in mucinous PCNs stratified by differentiation

**Figure 1:**
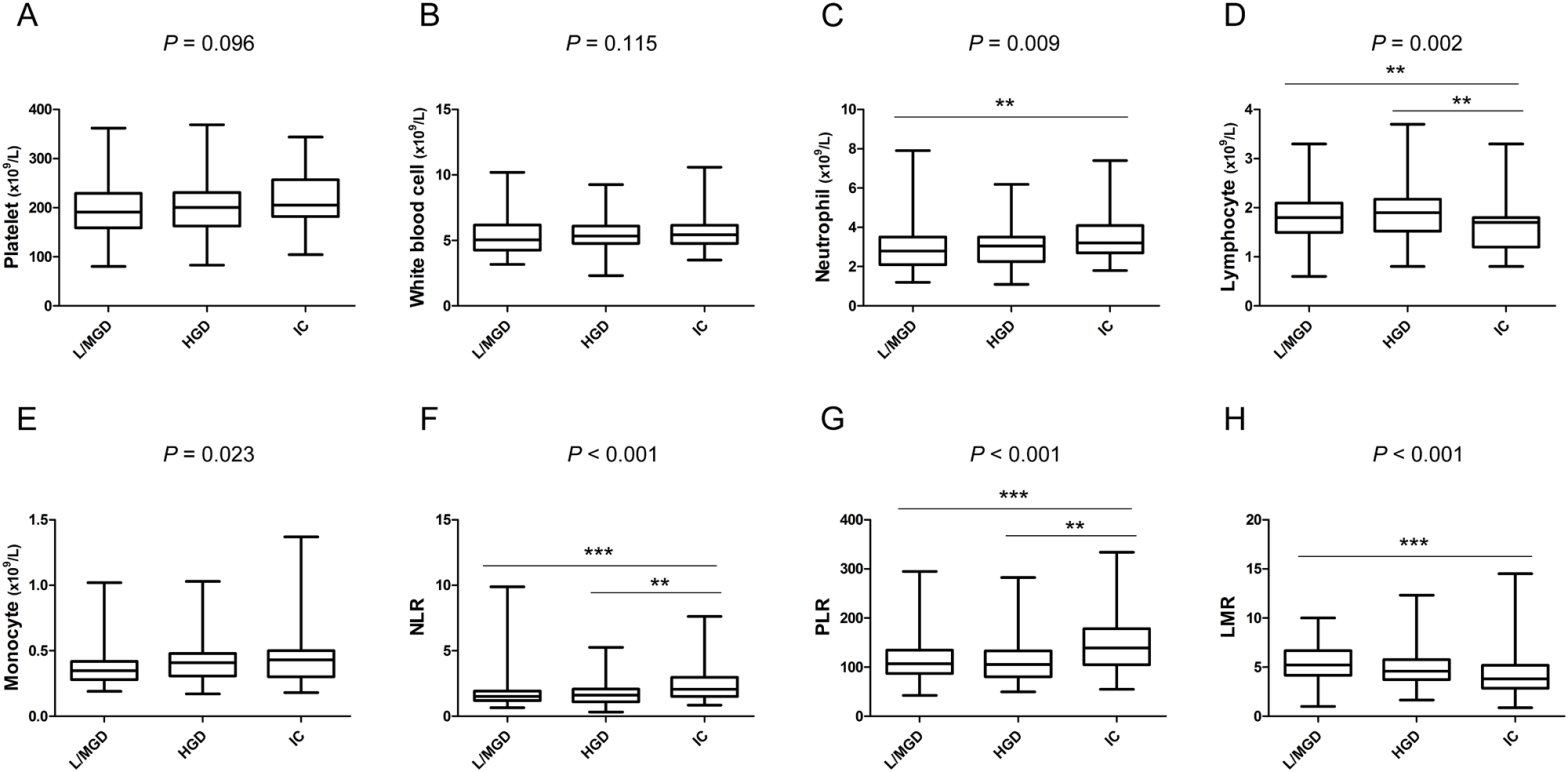
Box-plot diagrams were drawn according to the distribution of inflammatory markers in mucinous pancreatic cystic neoplasms stratified by pathology **(A)** platelet count; **(B)** white blood cell count; **(C)** neutrophil count; **(D)** lymphocyte count; **(E)** monocyte count; **(F)** neutrophil-to-lymphocyte ratio; **(G)** platelet-to-lymphocyte ratio; **(H)** lymphocyte-to-monocyte ratio. L/MGD, low-/moderate-grade dysplasia; HGD, high-grade dysplasia; and IC, invasive carcinomas. ^**^, *P* < 0.01; ^***^, *P* < 0.001.

Based on the histological differentiation of surgical resections, mucinous PCNs were divided into low-grade dysplasia (n = 94, 38.4%), moderate-grade dysplasia (n = 54, 22%), high-grade dysplasia (n = 48, 19.6%) and invasive carcinoma (n = 49, 20%). The first two were defined as benign lesions. The distribution of inflammatory markers in the various differentiation groups are shown in Figure 1. Except for platelet count, white blood cell count and monocyte count, all the other markers were distributed differently in these groups. Further comparisons of every two groups indicated that neutrophil count (*P* = 0.006), NLR (*P* < 0.001) and PLR (*P* < 0.001) were significantly elevated, whereas lymphocyte count (*P* = 0.006) and LMR (*P* < 0.001) were declined in invasive carcinomas compared with the low-/moderate-grade dysplasia group, respectively. Compared with the high-grade dysplasia group, the NLR (*P* = 0.006) and PLR (*P* = 0.001) were increased, and lymphocyte count (*P* = 0.003) was reduced in the invasive carcinoma group. However, none of the inflammatory factors displayed different distributions between the benign and high-grade dysplasia group.

### Univariate and multivariate analyses of predictive factors for invasive carcinoma

**Figure 2:**
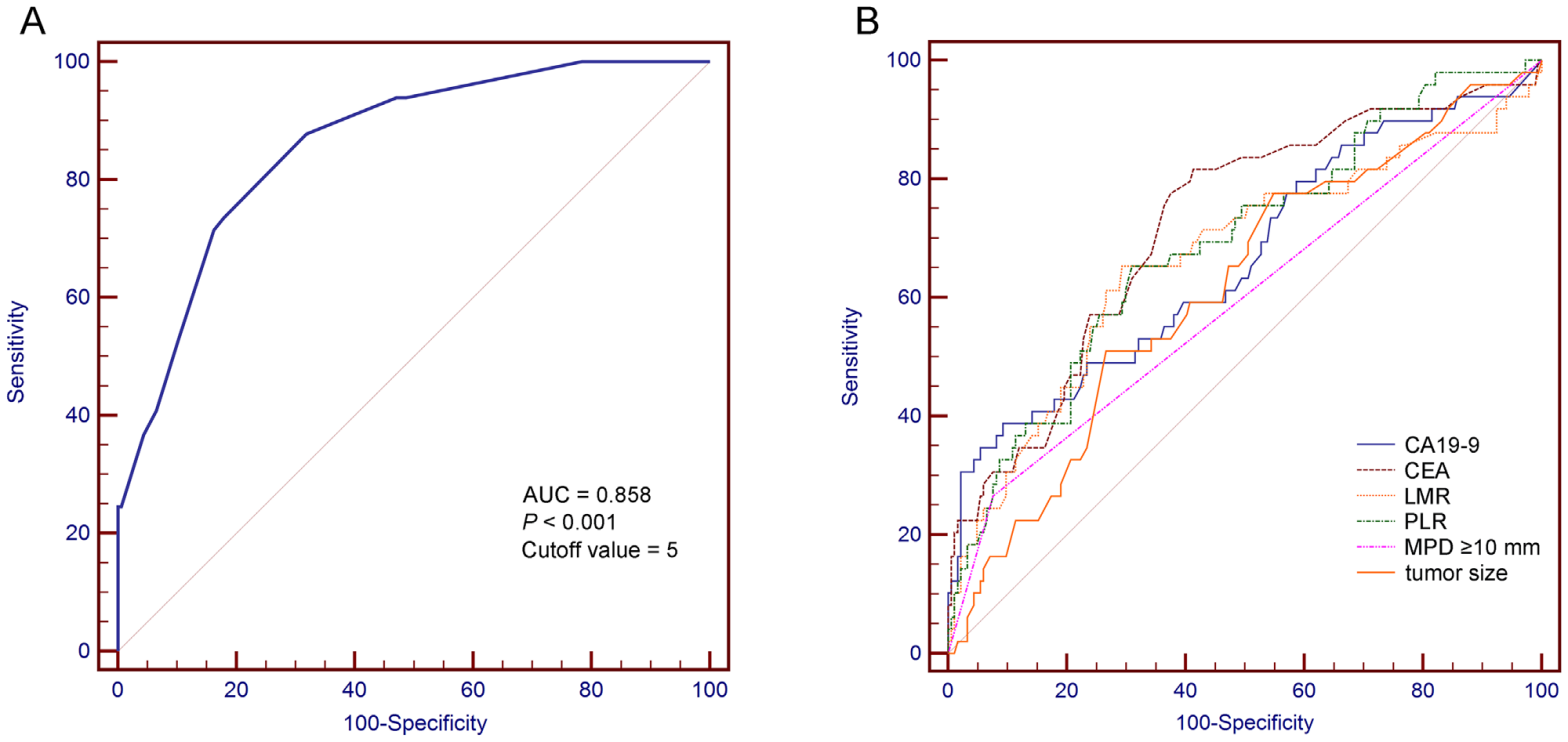
Receiver operating characteristic curves of **(A)** the predictive index score and **(B)** the independent predictors in detecting invasive carcinomas. AUC, area under the curve; MPD, main pancreatic duct; CA19-9, carbohydrate antigen 19-9; CEA, carcinoembryonic antigen; PLR, platelet-to-lymphocyte ratio; and LMR, lymphocyte-to-monocyte ratio.

The optimal cut-off values of these inflammatory markers were calculated via the ROC curves. Univariate analyses indicated that neutrophil count ≥ 2.8 × 10^9^/L (*P* = 0.024), lymphocyte count ≤ 1.9 × 10^9^/L (*P* < 0.001), PLR ≥ 125 (*P* < 0.001), NLR ≥ 1.96 (*P* < 0.001), and LMR ≤ 4.29 (*P* < 0.001) were significantly associated with mucinous PCN-derived invasive carcinoma (Table 2). In addition, patients who were over 61 years old (*P* = 0.014) and presented with symptoms (*P* = 0.013) were more prone to developing cancer. Radiological parameters of tumor size ≥ 49 mm (*P* = 0.003) and main pancreatic duct (MPD) diameter ≥ 10 mm (*P* < 0.001) also showed their predictive utilities in detecting invasive malignancy. In addition, of the 49 invasive carcinoma cases, 42 were derived from IPMN, and 7 were from MCN. The IPMN had a much higher chance than the MCN to turn into invasive cancer (25.9% vs. 8.4%, respectively, *P* = 0.001). Further multivariate analyses demonstrated that tumor size ≥ 49 mm (*P* = 0.002), MPD diameter ≥ 10 mm (*P* = 0.005), CA19-9 ≥ 42.7 U/mL (*P* = 0.005), CEA ≥ 1.9 ng/mL (*P* = 0.002), PLR ≥ 125 (*P* = 0.021), and LMR ≤ 4.29 (*P* = 0.001) were independent predictors of invasive carcinoma (Table 3). The AUC of the ROC curves of these independent factors including tumor size ≥ 49 mm, MPD diameter ≥ 10 mm, CA19-9 ≥ 42.7 U/mL, CEA ≥ 1.9 ng/mL, PLR ≥ 125, and LMR ≤ 4.29 curves were 0.617, 0.595, 0.664, 0.722, 0.691 and 0.667, respectively (Figure 2B).

**Table 2:** Univariate analyses of predictors of invasive carcinomas derived from mucinous pancreatic cystic neoplasms

Characteristic, n (%)	Low-/moderate-grade dysplasia (n = 148)	High-grade dysplasia (n = 48)	Invasive carcinoma (n = 49)	Non-invasive vs invasive, *P*
Age ≥ 61 years	44 (29.7%)	34 (70.8%)	29 (59.2%)	0.014
Male	43 (29.1%)	29 (60.4%)	24 (49.0%)	0.116
Symptom, present	62 (41.9%)	23 (47.9%)	31 (63.3%)	0.013
Tumor size ≥ 49 mm	45 (30.4%)	11 (22.9%)	25 (51.0%)	0.003
MPD diameter ≥ 10 mm	6 (4.1%)	9 (18.8%)	13 (26.5%)	< 0.001
Pathology				0.001
IPMN	73 (49.3%)	47 (97.9%)	42 (85.7%)	
MCN	75 (50.7%)	1 (2.1%)	7 (14.3%)	
IPMN classification				0.014
Main duct	20 (27.4%)	17 (36.2%)	18 (42.9%)	
Mixed type	12 (16.4%)	11 (23.4%)	12 (28.6%)	
Branch duct	36 (49.3%)	15 (31.9%)	7 (16.7%)	
Unknown	5 (6.8%)	4 (8.5%)	5 (11.9%)	
Albumin ≤ 39 g/L	50 (33.8%)	24 (50.0%)	25 (51.0%)	0.091
CA19-9 ≥ 42.7 U/mL^*^	12 (8.6%)	6 (12.8%)	19 (38.8%)	< 0.001
CEA ≥ 1.9 ng/mL^#^	55 (39.9%)	29 (61.7%)	40 (81.6%)	< 0.001
Neutrophil count ≥ 2.8 ×10^9^/L	77 (52.0%)	28 (58.3%)	35 (71.4%)	0.024
Lymphocyte count ≤ 1.9 ×10^9^/L	59 (39.9%)	22 (45.8%)	7 (14.3%)	< 0.001
PLR ≥ 125	45 (30.4%)	15 (31.3%)	32 (65.3%)	< 0.001
NLR ≥ 1.96	34 (23.0%)	12 (25.0%)	28 (57.1%)	< 0.001
LMR ≤ 4.29	41 (27.7%)	16 (33.3%)	32 (65.3%)	< 0.001

MPD, main pancreatic duct; IPMN, intraductal papillary mucinous neoplasm; MCN, mucinous cystic neoplasm; CA19-9, carbohydrate antigen 19-9; CEA, carcinoembryonic antigen; WBC, white blood cell; PLR, platelet-to-lymphocyte ratio; NLR, neutrophil-to-lymphocyte ratio; LMR, lymphocyte-to-monocyte ratio; ^*^, n = 235; ^#^, n = 234.

**Table 3:** Multivariate analyses of predictors for invasive carcinoma and predictive index score according to the odds ratio (n = 234)

Characteristic	Odds ratio	95% CI	*P*	Predictive index score
Tumor size ≥ 49 mm	3.852	1.670-8.882	0.002	3
MPD diameter ≥ 10 mm	4.565	1.578-13.205	0.005	4
CA19-9 ≥ 42.7 U/mL	3.704	1.482-9.255	0.005	3
CEA ≥ 1.9 ng/mL	3.967	1.665-9.452	0.002	3
PLR ≥ 125	2.538	1.149-5.604	0.021	2
LMR ≤ 4.29	3.857	1.695-8.775	0.001	3

MPD, main pancreatic duct; CA19-9, carbohydrate antigen 19-9; CEA, carcinoembryonic antigen; PLR, platelet-to-lymphocyte ratio; LMR, lymphocyte-to-monocyte ratio; CI, confidence interval.

### Construction and validation of the predictive model

All the above independent predictors were enrolled to establish the predictive model for invasive malignancy. The value of each variable was assigned based on the odds ratio (Table 3), and a point of 0 was allotted if a predictive factor was not possessed. The predictive index score = 3 (if tumor size ≥ 49 mm) + 4 (if MPD diameter ≥ 10 mm) + 3 (if CA19-9 ≥ 42.7 U/mL) + 3 (if CEA ≥ 1.9 ng/mL) + 2 (if PLR ≥ 125) + 3 (if LMR ≤ 4.29). The median of the predictive index score was 5 with a range between 0 and 18.

The predictive value of the model was assessed by using the ROC curve. A point of 5 was calculated as the optimal predictive index score with a sensitivity of 87.76% and a specificity of 68.11%. The AUC of this model was 0.858 (Figure 2A). The ROC curve comparison analyses showed that the predictive model had a much a better detecting ability than any individual predictors (Table 4).

**Table 4:** ROC curves comparison analyses of predictive index score and independent predictors (n = 234)

ROC curves (predictive index score vs.)	ΔAUC	SE	95% CI	Z statistic	*P* value
CA19-9	0.194	0.050	0.096-0.292	3.870	< 0.001
CEA	0.136	0.041	0.056-0.216	3.322	< 0.001
LMR	0.191	0.044	0.057-0.277	4.333	< 0.001
PLR	0.167	0.040	0.089-0.335	5.035	< 0.001
MPD ≥ 10 mm	0.263	0.035	0.195-0.332	7.517	< 0.001
Tumor size	0.241	0.048	0.090-0.245	4.201	< 0.001

ROC, receiver operating characteristic; AUC, area under the curve; SE, standard error; CI, confidence interval; MPD, main pancreatic duct; CA19-9, carbohydrate antigen 19-9; CEA, carcinoembryonic antigen; PLR, platelet-to-lymphocyte ratio; LMR, lymphocyte-to-monocyte ratio.

## DISCUSSION

Given the malignant potential of mucinous PCNs, the precise selection of patients who will benefit from surgical resections and those who will benefit from close follow-ups is crucial but difficult. Mainly, and based on imaging characteristics, high-risk signs for malignancies in pancreatic cysts including enhanced solid component, MPD size ≥ 10 mm, and obstructive jaundice were presented as surgical indications by the current international consensus guidelines. However, the sensitivity and specificity of these criteria in predicting high-grade dysplasia and invasive carcinoma varied largely in many retrospective cohorts, and the accuracy was not satisfied by most authors [18–21]. Thus, the investigations of appropriate diagnostic biomarkers have been conducted.

Recently, there has been a great interest in the inflammatory markers NLR and PLR in detecting malignant transformed mucinous PCNs. In our study, we analyzed the distributions of NLR and PLR in mucinous PCNs with different degrees of dysplasia. The results showed that both NLR and PLR were significantly elevated in patients with invasive carcinomas. The univariate analyses suggested that NLR ≥ 1.96 and PLR ≥ 125 were predictive markers of mucinous PCN-derived invasive carcinoma. In the multivariate analyses, only PLR was an independent predictor of invasive cancer, which was in accordance with the results from a report of Goh et al. [22]. The major differences were that some confounding factors regarding the selection of patients were excluded, and a doubled sample size was involved in our research. However, Gemenetzis et al. [17] and Arima et al. [23] reported that a high NLR rather than PLR was an independent predictor for invasive cancers in pancreatic cysts, respectively. This distinction could be explained in that only IPMN patients were enrolled in their studies. In clinical practice, the differential diagnosis between IPMN and MCN by preoperative imaging tests was limited even by endoscopic ultrasound and cyst fluid analysis [24].

An elevated PLR could be a consequence of an increased number of platelets, a decreased lymphocyte amount or both. Possible explanations regarding the relationship between PLR and pancreatic carcinogenesis are described in several other studies. The release of cytokines such as IL-1, IL-3 and IL-6 in the proinflammatory phase can stimulate the activation of megakaryocytes, which results in thrombocytosis [25]. However, growth factors, including platelet-derived growth factor, platelet factor 4 and TGF-β generated by platelets, can promote the proliferation and invasion of tumor cells [26, 27]. Apart from inflammation, the suppression of the lymphocyte-mediated immune response by inhibitory mediators such as IL-10 and TGF-β also plays a crucial role in the progression of pancreatic carcinomas [28, 29]. Thus, a high circulating cell-based PLR may be a magnified reflection of both the inflammatory and immune response in the regional tumor microenvironment. Additionally, another inflammatory index of LMR demonstrated the independent predictive role of invasive carcinoma in our cohort. To our understanding, this is the first research investigating LMR in pancreatic cystic neoplasms. Based on our findings, we provide a novel serum marker for the individual patient’s risk assessment at the time of PCN diagnosis. Previous studies have reported that local tumor-associated macrophages mainly recruited from circulating monocytes could exert immunosuppressive influences on facilitating tumor growth and angiogenesis [30]. Thus, LMR undertakes a similar indicative role of the complex tumor microenvironment as that of PLR. The independent predictive value of both PLR and LMR in identifying invasive carcinomas provides a certain support for making therapeutic decisions regarding mucinous PCNs.

We further evaluated the predictive abilities of these inflammatory biomarkers by using ROC curves, and the AUC of PLR and LMR were 0.691 and 0.667, respectively. The results indicated that both PLR and LMR could not serve as ideal diagnostic markers of mucinous PCN patients harboring invasive cancers. To establish an effective predictive model for mucinous PCN-derived malignancies, four other independent factors including tumor size ≥ 49 mm, MPD diameter ≥ 10 mm, CA19-9 ≥ 42.7 U/mL and CEA ≥ 1.9 ng/mL were incorporated. The AUC of our predictive model was 0.858, which showed a significantly superior performance in detecting malignancy than any individual components. The optimal predictive value of this model was 5 with a sensitivity of 87.76% but a relatively low specificity of 68.11%, which would bring about unnecessary resections of benign lesions. However, in our study, only 24 (27.6%) of the cases without invasive cancers but that scored ≥ 5 were branch-duct IPMNs (BD-IPMNs); all other types of mucinous PCNs were recommended for surgical resections due to the high risk of malignant transformation by the guidelines. In addition, 10 of the 24 BD-IPMNs were pathologically confirmed with high-grade dysplasia, and surgical interventions were imperative for these cases. In general, since 4 serum markers and 2 cross-sectional imaging-based features are collected from routinely preoperative assessments, our predictive model is easily accessible and helpful for clinicians to identify malignancy in patients with mucinous PCNs.

There are several limitations that are necessary to mention. As a retrospective cohort of resected patients, the true predictive value of these inflammatory factors could not be evaluated mainly due to the lack of follow-up cases. Furthermore, peripheral blood cells can be affected by various factors, and the detecting utility of the inflammatory markers is limited, particularly in patients with infectious diseases or undergoing invasive tests. In addition, no correlation between the biomarkers and high-grade dysplasia was discovered in our study. The search for novel diagnostic strategies for mucinous PCNs with high-grade dysplasia is urgent, since patients at this phase of PCN can greatly benefit from surgical therapies.

## MATERIALS AND METHODS

Data of 276 patients who underwent surgical resections for mucinous pancreatic cystic neoplasms in Zhong Shan Hospital, Fudan University, from February 2008 to April 2017 were retrospectively evaluated. Patients whose records for preoperative blood cell counts were unavailable, had a history of malignancy or hematological diseases, exhibited infectious diseases or received invasive procedures within two weeks before the operations were excluded (Supplementary Figure 1).

Demographic, clinical and pathological data were collected from the medical records of the selected patients. Routine laboratory tests for albumin, CA19-9, CEA, platelet count, white blood cell count, neutrophil count, lymphocyte count, and monocyte count were performed prior to surgery with a median time of 4 days (IQR 2-5). The NLR and PLR were defined as the ratio of the absolute neutrophil count and platelet count divided by the absolute count of lymphocyte, respectively. Similarly, LMR was calculated as the absolute lymphocyte count divided by the absolute monocyte count. The diameters of the cystic tumor and main pancreatic duct were measured according to the images of the CT or MRI. The largest lesion was evaluated if multifocal tumors existed. All the cases were confirmed by the final pathology of the resected specimens. The tumor differentiation was classified based on the most aggressive histological changes, including low-grade dysplasia, moderate-grade dysplasia, high-grade dysplasia and invasive carcinomas.

This research was approved by the Ethics Committee of Zhong Shan Hospital, and written informed consent was obtained from all the patients.

### Statistical analysis

Continuous variables presented with a median and interquartile range, while categorical variables were described as a whole number and percentage. The distributions of continuous variables among multiple independent groups were compared by the Kruskal-Wallis test. Univariate analyses of categorical variables between two independent groups were performed using the chi-square test. Multivariate analyses were conducted to assess the independent predictors of invasive carcinomas by the logistic regression model with the forward elimination method (likelihood-ratio test). All the above statistical analyses were performed on SPSS version 19.0 software (IBM Corp., Armonk, NY, USA). Receiver operating characteristic (ROC) curves were calculated to determine the optimal cut-off values of the continuous variables. The area under the curve (AUC) of the ROC curves was compared using the method of DeLong et al. [31] with MedCalc version 11.4.2.0 software (MedCalc Software bvba, Ostend, Belgium). A two-sided *P* value of less than 0.05 was defined as significant.

## CONCLUSION

The systemic inflammation response is closely related to the progression of mucinous pancreatic cysts. Circulating cell-based biomarkers PLR and LMR have been demonstrated as the independent predictors of mucinous PCN patients harboring invasive carcinomas. Our predictive model is reliable in identifying invasive malignancies and can be used as a convenient tool for the preoperative assessments of pancreatic cystic neoplasms, and all the indexes are easily extracted from routine tests. However, the exact mechanisms regarding the interaction between inflammation and mucinous PCN are still unknown, and anti-inflammatory therapies may slow down the disease course or even prevent malignant changes in pancreatic cysts, which need to be clarified in the future.

## SUPPLEMENTARY MATERIALS FIGURES AND TABLES



## Abbreviations

PCN: pancreatic cystic neoplasm
NLR: neutrophil-to-lymphocyte ratio
PLR: platelet-to-lymphocyte ratio
LMR: lymphocyte-to-monocyte ratio
ROC: receiver operating characteristic
AUC: area under the curve
IPMN: intraductal papillary mucinous neoplasm
MCN: mucinous cystic neoplasm
IQR: interquartile range
MPD: main pancreatic duct
CA19-9: carbohydrate antigen 19-9
CEA: carcinoembryonic antigen
BD-IPMN: branch-duct IPMN
MRI: magnetic resonance imaging
CT: computed tomography
