# Supplementary material for: The value of systemic inflammatory markers in identifying malignancy in mucinous pancreatic cystic neoplasms
Source: Oncotarget. 2017 Dec 14;8(70):115561–9. doi: 10.18632/oncotarget.23310 (PMC5777793; doi:10.18632/oncotarget.23310)
Supplement: Supplementary file 1 [file oncotarget-08-115561-s001.pdf]

The value of systemic inflammatory markers in identifying malignancy in mucinous pancreatic cystic neoplasms

SUPPLEMENTARY MATERIALS

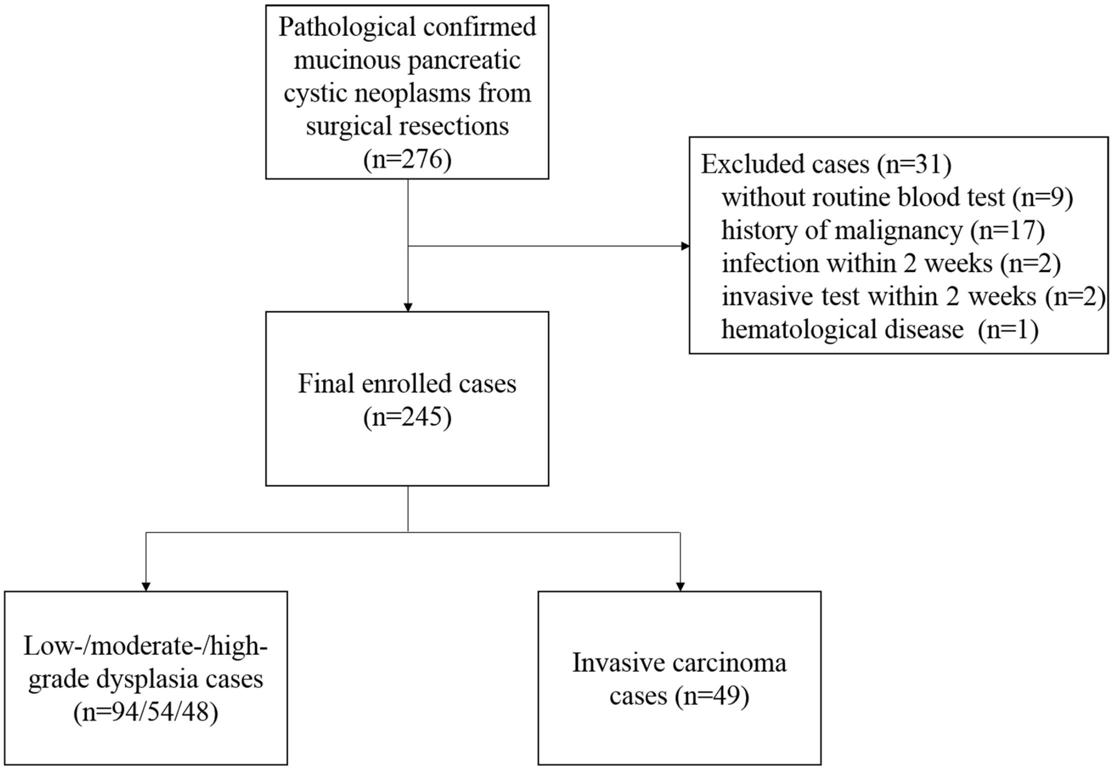

Supplementary Figure 1: Flow chart of the case selection process.
